# When ease of use is not enough: self-efficacy and continued use of AI educational tools in resource-constrained universities

**DOI:** 10.3389/fpsyg.2026.1896153

**Published:** 2026-07-22

**Authors:** Yao Wan, Kuanmin Lu

**Affiliations:** 1 School of Marxism, Xi’an University of Technology, Xi’an, Shaanxi, China; 2 School of Marxism, Xi’an International Studies University, Xi’an, Shaanxi, China

**Keywords:** AI educational tools, continuance intention, generative artificial intelligence, resource-constrained universities, self-efficacy

## Abstract

**Purpose:**

Generative artificial intelligence (AI) is increasingly used in higher education, yet students’ continued use of AI educational tools may operate differently in resource-constrained university contexts. This study examines college students’ continuance intention to use AI educational tools in local undergraduate universities across five northwestern Chinese provinces and develops a capability-centric extension of the unified theory of acceptance and use of technology (UTAUT).

**Methods:**

A sequential explanatory mixed-methods design was adopted. After pilot-based construct purification, 703 valid survey responses from students with prior AI tool experience were analyzed using partial least squares structural equation modeling (PLS-SEM). Additional analyses included common method bias diagnostics, PLSpredict, cross-validated predictive ability testing, multi-group analysis, and post-hoc power analysis. Semi-structured interviews with 25 students were used to contextualize the quantitative findings.

**Results:**

Self-efficacy was the strongest predictor of continuance intention, followed by performance expectancy, perceived policy signals, and social influence. Algorithmic trust showed only a weak positive association, whereas effort expectancy showed a weak but significant negative association, contrary to the original UTAUT prediction. Qualitative findings indicated that students perceived AI tools as easy to access but cognitively demanding to use effectively, due to prompt refinement, factual verification, error correction, and task adaptation.

**Conclusion:**

Continued AI educational tool use in resource-constrained universities depends less on surface-level ease of use and more on students’ perceived capability to manage the full AI-assisted learning workflow. This study extends UTAUT-based AI adoption research by foregrounding self-efficacy and proposing a capability-centric perspective on generative AI continuance in higher education.

## 1 Introduction

Generative artificial intelligence has become increasingly visible in higher education, where students use AI tools for information search, writing support, translation, coding assistance, and problem solving. Yet its promotion and practice at local undergraduate universities in five northwestern Chinese provinces differ sharply from resource-rich universities in eastern China. Restricted by regional fiscal investment, digital infrastructure and students’ baseline digital literacy, these universities face inadequate resource supply. Such shortages emerge in unstable campus networks, insufficient intelligent teaching facilities, limited access to paid AI tools, and scarce training on AI operation.

Existing studies have begun to examine regional disparities in Chinese college students’ adoption of generative AI (Jiang et al., 2025). Still, most research relies on convenient samples from central cities and well-resourced universities. Few empirical studies specifically target local undergraduate schools in Northwest China where educational resources fall short. Against this backdrop, students’ sustained use of AI educational tools depends on more than perceived ease of use. It also hinges on their ability to verify AI-generated content, refine prompts, correct flawed outputs, and maintain consistent AI learning amid insufficient institutional policies and training support.

Moreover, educational applications of generative AI may widen the new digital divide. Beckman et al. (2025) coined the term “GenAI divide” among college students. This divide covers not only access to AI tools but also AI literacy, usage confidence, institutional support, and the capacity to translate AI into academic advantages. Thus, insufficient resource supply is not merely a problem of tool accessibility. It may also hinder students’ development of effective, sustained, high-quality AI-assisted learning practices. This study targets local undergraduate universities across five northwestern provinces. It explores the formative mechanisms underlying college students’ sustained intention to use AI educational tools.

Three critical gaps limit empirical tests of the above predictions in current literature:

First, existing samples remain geographically and institutionally narrow. Research on generative AI acceptance in higher education mostly draws samples from resource-abundant universities in Beijing, Guangzhou and other eastern cities (Shahzad et al., 2024; Moradi, 2025). A small body of studies has explored east–west regional gaps, yet these only treat region as a control variable (Jiang et al., 2025). Few investigations focus on northwestern local undergraduate colleges with weak network infrastructure, limited hardware, restricted paid AI tool access and inadequate AI training. Findings derived from samples of well-resourced universities cannot be directly generalized across regions without further validation.

Second, some extended adoption models include constructs with unclear boundaries. Recent research incorporates personal innovativeness or exploratory tendencies into student generative AI acceptance frameworks to predict behavioral intentions toward tools such as ChatGPT (Strzelecki, 2024; Al Amin et al., 2026). In AI learning contexts, direct measurement of willingness to explore or sustain new technology creates conceptual overlap with continuance intention. In the pilot survey, personal innovativeness showed insufficient discriminant validity from continuance intention. It was therefore removed from the formal model to avoid construct redundancy. Instead of arbitrarily adding exploratory tendency variables, this study eliminates constructs with weak discriminant validity. It refocuses on theoretically distinct antecedents: performance expectancy, effort expectancy, social influence, self-efficacy, algorithmic trust, and perceived policy signals. This adjustment improves interpretive clarity and estimation robustness of the extended UTAUT model.

Third, quantitative models alone provide limited insight into how students actually experience AI use under resource constraints. Survey data can test associations among constructs, but they do not fully capture everyday difficulties such as reliance on incomplete free AI tools, repeated prompt adjustment, and the time required for manual verification. Qualitative evidence is therefore needed to contextualize the statistical findings.

To address these gaps, this study adopts a sequential explanatory mixed-methods design. It builds on the classic UTAUT model and expands the variable system to match dual contextual features: insufficient resource supply in Northwest China and generative AI characteristics. The study retains three core UTAUT antecedents: performance expectancy, effort expectancy, and social influence. It removes personal innovativeness due to poor discriminant validity. Three context-specific constructs are newly added: self-efficacy (SE), algorithmic trust (AT), and perceived policy signals (PPS). The sample only includes enrolled students with prior AI tool experience. This research exclusively examines long-term continuance intention rather than initial adoption among inexperienced users. Three core research questions guide the analysis:

- After removing redundant constructs, can the extended UTAUT model effectively explain college students’ continuance intention toward AI educational tools at resource-constrained northwestern universities?
- Which antecedents show stronger or weaker associations with BI, and how do these patterns support a capability-centric interpretation?
- How do resource shortages in Northwest China and cognitive costs of AI content verification provide contextual support for quantitative model correlations?

By answering these questions, this study contributes to technology acceptance research in two ways. First, it extends UTAUT to a less examined higher education context where institutional and digital resources are relatively limited. Second, it proposes a capability-centric perspective, suggesting that continued AI educational tool use depends not only on perceived usefulness or ease of use, but also on students’ perceived ability to manage the full process of AI-assisted learning, including prompt refinement, output verification, error correction, and task adaptation.

## 2 Theoretical background and hypotheses

### 2.1 Theoretical framework: extending UTAUT in resource-constrained university contexts

This study draws on the unified theory of acceptance and use of technology (UTAUT) as the theoretical foundation for explaining students’ continuance intention to use AI educational tools. The model retains three core UTAUT constructs: performance expectancy (PE), effort expectancy (EE), and social influence (SI). These constructs capture students’ perceptions of whether AI tools are useful for learning, whether they are easy to use, and whether important others encourage or normalize their use. In the context of generative AI-supported learning, however, these traditional technology acceptance factors may not fully capture the demands students face when using AI tools for academic purposes.

To reflect the specific features of AI educational tool use in resource-constrained university contexts, this study adds three context-relevant constructs: self-efficacy (SE), algorithmic trust (AT), and perceived policy signals (PPS). Self-efficacy refers to students’ perceived capability to complete learning tasks with AI tools, solve usage problems independently, verify and correct AI-generated outputs, and adapt AI responses to discipline-specific assignments. Algorithmic trust refers to students’ confidence in the accuracy, stability, and data security of AI educational tools. Perceived policy signals refer to students’ perceptions of whether their university communicates support for AI use through policies, classroom guidance, training activities, and learning support arrangements.

This study does not aim to reproduce the original UTAUT or UTAUT2 model in full. Instead, it adapts UTAUT to the present research focus: continuance intention among students who already have experience using AI educational tools. One important modeling decision concerns facilitating conditions (FC). In the original UTAUT framework, facilitating conditions refer to users’ perceptions of whether sufficient resources, technical support, infrastructure, and compatibility conditions are available for technology use. This construct is often treated as closely related to actual use behavior, especially when researchers examine whether users have the practical conditions needed to use a system. In the present study, however, the dependent variable is continuance intention rather than observed use behavior. In addition, the questionnaire did not comprehensively measure the full range of FC dimensions, such as network quality, device availability, platform compatibility, and technical service support. For this reason, FC was not included as a separate latent construct in the formal structural model.

Instead, resource constraints are treated as the contextual boundary of the study, and perceived policy signals are introduced to capture a more specific institutional process. PPS is related to the broader support environment, but it is conceptually different from FC. FC focuses on whether students have access to the resources and support needed to use a technology. PPS focuses on whether students perceive clear institutional messages that AI use is encouraged, legitimate, and supported in learning contexts. In resource-constrained universities, such policy signals may matter because students may not receive sufficient technical resources or systematic training. Even when material support is limited, visible institutional guidance, instructor encouragement, and training opportunities can reduce uncertainty and help students judge whether continued AI use is acceptable and worthwhile.

The model also excludes personal innovativeness, which was considered during the pilot stage. Although personal innovativeness is often used in technology acceptance research, pilot results in this study suggested substantial overlap between personal innovativeness and continuance intention. In this context, items measuring students’ willingness to actively explore or try new learning technologies were difficult to distinguish from their intention to continue using AI educational tools. To reduce construct redundancy and improve discriminant validity, personal innovativeness was removed from the formal model.

Overall, the final model includes six antecedents of continuance intention: performance expectancy, effort expectancy, social influence, self-efficacy, algorithmic trust, and perceived policy signals. This structure preserves the core logic of UTAUT while adapting it to the realities of generative AI learning in resource-constrained universities. It allows the study to examine not only whether students perceive AI tools as useful and easy to use, but also whether they feel capable of managing AI-generated outputs, whether they trust the tools sufficiently, and whether they perceive institutional signals that support continued AI use.

### 2.2 The capability-centric perspective

In resource-constrained university contexts, students’ continued use of AI educational tools may depend not only on whether they perceive the tools as useful or easy to use, but also on whether they feel capable of managing the learning process supported by these tools. Compared with students at well-resourced universities, students in local universities in Northwest China may have less stable access to digital infrastructure, fewer opportunities for systematic AI-related training, and more limited access to advanced or paid AI functions. Prior research has also shown that regional differences may shape Chinese college students’ intentions to adopt generative AI, with western universities facing greater challenges in digital infrastructure and AI learning support (Jiang et al., 2025). In such contexts, students often need to rely more heavily on their own abilities to select appropriate tools, formulate prompts, solve usage problems, and adapt AI-generated outputs to academic tasks.

The nature of generative AI further increases the importance of student capability. Although AI tools can provide instant explanations, writing support, translation, coding assistance, and personalized learning suggestions, their outputs are not always reliable. Large language models may produce hallucinated content, including factual errors, fabricated information, logical inconsistencies, and confident but inaccurate responses (Ji et al., 2023). Therefore, using AI educational tools for academic purposes is rarely a simple process of asking a question and accepting an answer. Students often need to evaluate the output, check facts, revise prompts, correct errors, and integrate the revised content into their coursework. Continued AI use thus requires not only basic operational skills, but also the ability to judge, verify, and adapt AI-generated information.

Based on these considerations, this study develops a capability-centric perspective to interpret students’ continuance intention toward AI educational tools. This perspective does not replace the core assumptions of UTAUT. Performance expectancy, effort expectancy, and social influence remain important for explaining students’ technology-related perceptions. However, in generative AI learning contexts, especially where institutional and digital resources are limited, students’ perceived capability may become particularly salient. Students may recognize that AI tools are useful, receive encouragement from teachers or peers, or perceive institutional support for AI use. Yet these factors may not translate into continued use unless students believe they can handle the practical and cognitive demands that arise during AI-assisted learning.

Self-efficacy is therefore central to the capability-centric perspective. In this study, self-efficacy is not limited to general confidence in using technology. It refers to students’ perceived ability to use AI tools for learning tasks, solve problems independently, identify inaccurate outputs, verify information, correct errors, and adapt AI-generated content to discipline-specific requirements. Recent studies on ChatGPT and generative AI adoption similarly suggest that self-efficacy plays an important role in shaping students’ willingness to use AI tools, because it strengthens their confidence in dealing with unfamiliar or uncertain AI-supported learning situations (Tailor and Tailor, 2025; Ursavaş et al., 2025).

Accordingly, the capability-centric perspective highlights a shift in what matters for continued AI educational tool use. In traditional technology acceptance settings, ease of use often reduces the effort required to operate a system. In generative AI learning, however, the visible ease of interaction may coexist with hidden demands for verification, correction, and task adaptation. For students in resource-constrained universities, these demands may become more consequential because external support is not always sufficient. Continued use may therefore depend less on simple operational ease and more on students’ confidence that they can manage the full process of AI-assisted learning.

**Figure 1:**
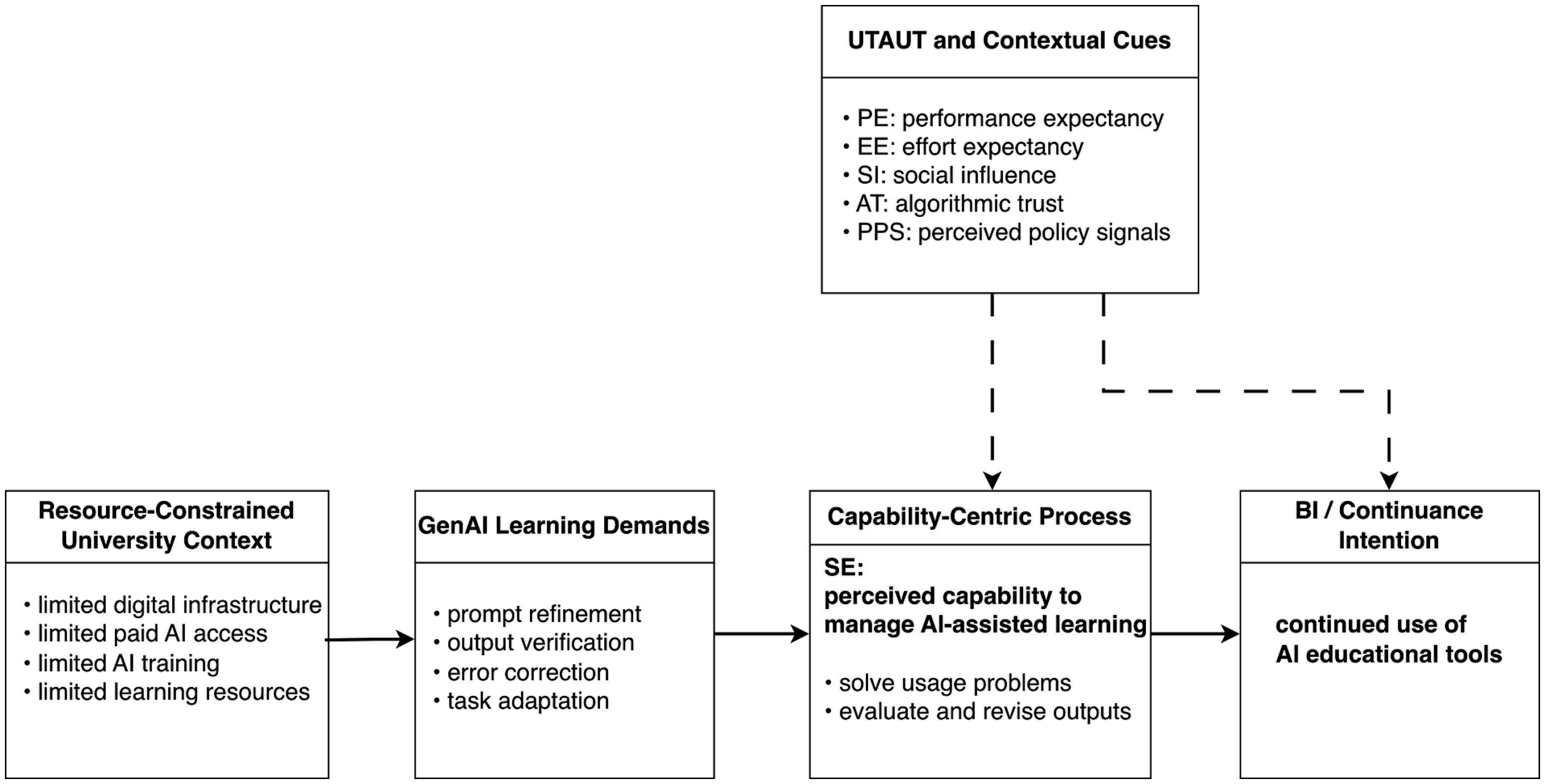
Conceptual explanatory framework: a capability-centric perspective on AI educational tool continuance.

Based on the above discussion, Figure 1 presents the conceptual explanatory framework of the capability-centric perspective. The framework illustrates how resource-constrained university contexts and GenAI learning demands make students’ perceived capability central to understanding continued AI educational tool use.

### 2.3 Hypotheses development

#### 2.3.1 Performance expectancy and continuance intention

Performance expectancy serves as a core UTAUT predictor of technology usage intention. It describes the degree to which individuals believe a technology improves task performance or academic outcomes (Venkatesh et al., 2003). In AI education contexts, performance expectancy reflects perceptions that AI tools boost learning efficiency, streamline coursework, and optimize information retrieval and knowledge comprehension. Existing ChatGPT and generative AI adoption studies confirm that students form stronger usage intentions when they perceive AI tools enhance academic performance, save study time, or improve assignment quality (Strzelecki, 2024; Shahzad et al., 2024). For students at resource-constrained northwestern undergraduate universities, AI tools compensate for inadequate academic support through instant feedback and personalized tutoring. This instrumental value readily translates into sustained usage intentions. The following hypothesis is proposed:

*H1*: PE positively predicts college students’ continuance intention to use AI educational tools.

#### 2.3.2 Effort expectancy and continuance intention

Effort expectancy measures the perceived ease or difficulty of using a technology, including system learnability, operability, and interface clarity (Venkatesh et al., 2003). In the classic UTAUT framework, users are more likely to form favorable usage intentions when a technology is easy to learn and operate. Existing ChatGPT adoption research similarly treats perceived ease of use or effort expectancy as an important predictor of students’ acceptance of generative AI tools, because lower operational barriers can reduce psychological resistance to technology engagement (Shahzad et al., 2024; Strzelecki, 2024).

However, perceived ease of use may be more complex in generative AI learning contexts than in conventional learning management systems. Although natural language interfaces lower the initial threshold for interaction, effective academic use often extends beyond submitting prompts and receiving outputs. AI-generated responses may contain factual errors, logical gaps, fabricated citations, or misleadingly confident statements. Students may therefore need to refine prompts, verify outputs, correct misinformation, and adapt AI-generated content to discipline-specific coursework (Ji et al., 2023; Walters and Wilder, 2023).

It should be noted that EE in this study was measured using traditional ease-of-use items and should not be treated as a direct measure of implicit cognitive cost. Instead, qualitative interviews are used to contextualize whether students’ experiences of post-generation verification and revision help explain any unexpected EE path direction. Following the classic UTAUT prediction, the corresponding hypothesis is stated below:

*H2*: EE positively predicts college students’ continuance intention to use AI educational tools.

#### 2.3.3 Social influence and continuance intention

Social influence captures perceptions that significant others endorse technology adoption, including instructors, peers, institutional bodies, and learning communities (Venkatesh et al., 2003). University AI tool use is not purely individual decision-making. It is shaped by curriculum requirements, peer usage norms, disciplinary standards, and institutional attitudes. When instructors encourage rational AI integration or peer groups widely deploy AI for literature searches, drafting assistance and coursework completion, students view AI as academically legitimate and develop stronger continuance intentions.

Existing generative AI adoption literature identifies social influence as a meaningful explanatory variable, though its magnitude varies across cultural contexts, learner autonomy levels, and risk perceptions (Strzelecki, 2024; Ursavaş et al., 2025). For northwestern local undergraduate students, institutional and instructor attitudes toward AI carry disproportionate weight. Amid limited campus resources, students rely heavily on external social norms and peer experiences to judge whether sustained AI engagement merits investment. The hypothesis below is advanced:

*H3*: SI positively predicts college students’ continuance intention to use AI educational tools.

#### 2.3.4 Self-efficacy and continuance intention

Self-efficacy refers to individuals’ confidence in completing targeted tasks and overcoming technical usage barriers. Effective generative AI learning demands more than basic technical operation. It requires crafting effective prompts, refining query wording, identifying factual inaccuracies in AI outputs, verifying information authenticity, and revising generated content to fit assignment requirements. This study’s operationalization of self-efficacy moves beyond generic technical confidence. It captures students’ perceived capacity to oversee complete AI-assisted learning workflows within resource-constrained university environments.

Prior empirical work confirms self-efficacy as a vital psychological antecedent of generative AI tool adoption. Ursavaş et al. (2025) found self-efficacy significantly predicts college students’ behavioral intentions to use GenAI, highlighting the central role of ability judgments in AI acceptance. At northwestern local undergraduate universities, students lack consistent formal training, technical support, and premium AI tool access. Sustained AI learning therefore depends on independent problem-solving, content verification, and task adaptation capabilities. Higher self-efficacy strengthens student confidence in navigating AI uncertainty and maintaining consistent usage. The following hypothesis is formulated:

*H4*: SE positively predicts college students’ continuance intention to use AI educational tools.

#### 2.3.5 Algorithmic trust and continuance intention

Algorithmic trust describes students’ subjective beliefs regarding the accuracy, system stability, data privacy security, and decision reliability of AI educational tools. Educational generative AI application relies heavily on student evaluations of output quality. Students prioritize sustained AI integration into daily study routines when they perceive reliable, accurate learning support. Fears of AI factual errors, privacy leakage, and uncontrollable outputs weaken continuance intentions.

Existing scholarship identifies trust as a foundational factor shaping ChatGPT and generative AI acceptance. Shahzad et al. (2024) incorporated trust as a core construct in their Chinese student ChatGPT adoption model, demonstrating trust’s impact on perceived ease of use, usefulness, and perceived intelligence. Generative AI’s inherent hallucination risks further underscore algorithmic trust’s relevance in educational environments (Ji et al., 2023; Walters and Wilder, 2023). This view is consistent with broader research showing that human trust in AI systems depends on perceived reliability, transparency, and task risk (Glikson and Woolley, 2020). Therefore, greater student confidence in AI accuracy, stability, and security is expected to correlate with stronger continuance intentions. The corresponding hypothesis is proposed:

*H5*: AT positively predicts college students’ continuance intention to use AI educational tools.

#### 2.3.6 Perceived policy signals and continuance intention

Perceived policy signals measure student awareness of institutional generative AI promotion policies, classroom advocacy, training programs, and learning support resources. Unlike UTAUT’s facilitating conditions construct, which emphasizes resource availability and technical support infrastructure, perceived policy signals center on institutional communications that legitimize AI learning through formal policies, instructor guidance, and training events. At resource-constrained universities, institutions cannot immediately provide adequate hardware, paid AI subscriptions, and full technical support. Clear policy advocacy, instructor guidance, and limited training opportunities nevertheless reduce student normative uncertainty and justify sustained AI investment.

Existing research establishes that institutional policy, training, ethical guidance, and communication shape university generative AI adoption beyond individual cognitive factors. Jin et al. (2025) analyzed global institutional GenAI policies and guidelines, documenting university strategies of ethical frameworks, training programs, assessment adjustments, and transparent communication to integrate generative AI into teaching. Nikolic et al. (2024) similarly noted that institutional training, policy, and comprehensive support systems drive academic staff and student GenAI uptake. For northwestern local undergraduate students, policy signals deliver institutional legitimacy and directional guidance alongside tangible resource support. Stronger perceived institutional signals correlate with higher continuance intentions toward AI educational tools. The hypothesis below is proposed:

*H6*: PPS positively predicts college students’ continuance intention to use AI educational tools.

**Figure 2:**
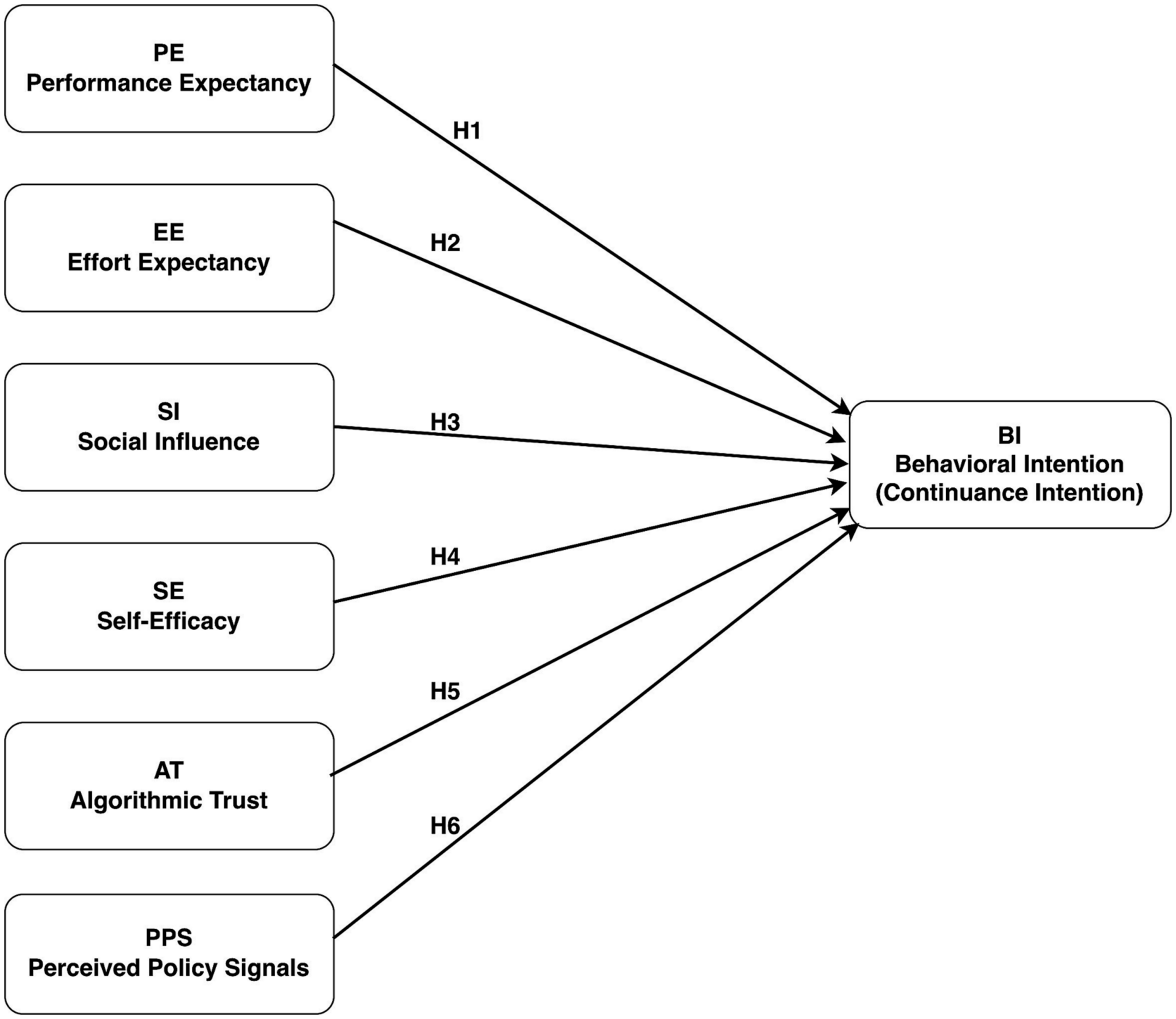
Empirical research model.

Based on the preceding theoretical discussion and hypotheses, the empirical research model is presented in Figure 2.

## 3 Materials and methods

### 3.1 Study design

This research employs a sequential explanatory mixed-methods design. Three sequential phases refine measurement scales, test structural models, and interpret mechanisms through contextual qualitative evidence.

Pilot survey: Validate item readability, construct reliability, and discriminant validity. Sample *N* = 286.

*Main questionnaire survey*: Test measurement models, structural path relationships, predictive validity, and multi-group heterogeneity via PLS-SEM, PLSpredict, and CVPAT. Sample *N* = 703 after data cleaning.

*Semi-structured interviews*: Extract thematic contextual evidence to interpret quantitative path results, focusing on resource scarcity, AI content verification costs, self-efficacy, and perceived policy signals. Interview sample *N* = 25.

The pilot survey identified severe discriminant validity issues for the personal innovativeness construct. Its HTMT value against continuance intention reached 0.931, exceeding standard cutoffs for distinguishable latent variables. To purify measurement constructs and eliminate overlapping variance, personal innovativeness was excluded from the formal structural model. The finalized model retains six antecedents: PE, EE, SI, SE, AT, and PPS.

The main questionnaire collected 852 raw responses. Data cleaning removed low-quality submissions, yielding 703 valid samples for PLS-SEM analysis. Twenty-five students with AI learning experience completed follow-up semi-structured interviews to contextualize significant quantitative paths.

### 3.2 Participants and sampling

Survey participants were enrolled undergraduate students with prior experience using AI educational tools at local universities across five northwestern Chinese provinces: Shaanxi, Gansu, Qinghai, Ningxia, and Xinjiang. The questionnaire first defined AI educational tools, including ChatGPT, DeepSeek, Wenxin Yiyan, iFlytek Spark, Doubao, Claude, and other generative AI platforms supporting thesis drafting, literature retrieval, language polishing, code debugging, and academic problem-solving. A screening question captured AI usage experience with five response tiers: never used, less than 1 month, 1–3 months, 3–6 months, and 6 months or longer. Respondents selecting “never used” completed an open-ended question on non-adoption reasons and exited the survey. Only participants with prior AI experience completed the full scale, as this study exclusively investigates continuance intention rather than initial adoption.

Stratified sampling selected eight local undergraduate universities across the five northwestern provinces. At least 100 questionnaires were distributed per institution. Sampling balanced regional distribution, institutional type, grade levels, and disciplinary backgrounds to mitigate sample bias concentrated in single schools or majors. The study focuses on northwestern local undergraduate colleges to address literature gaps: most generative AI adoption research draws samples from central cities and eastern resource-rich universities, ignoring infrastructure, training, and paid tool access disparities in western China.

Regional educational resource disparities are contextualized via official 2024 provincial education expenditure statistics published by the China Education Economic Information Network. Table 1 compares total educational expenditure and fiscal educational funding between five northwestern provinces and three representative eastern provinces (Jiangsu, Zhejiang, Guangdong).

**Table 1:** 2024 Educational expenditure comparison: five northwestern provinces vs. three eastern provinces.

Region	Included provinces	Total educational expenditure (Billion RMB)	Fiscal educational funding (Billion RMB)	Comparative note
Five Northwestern Provinces	Shaanxi, Gansu, Qinghai, Ningxia, Xinjiang	509.127	424.678	Baseline reference group
Three Eastern Provinces	Jiangsu, Zhejiang, Guangdong	1,465.839	1,109.009	Total expenditure = 2.88 × northwest total; fiscal funding = 2.61 × northwest total

Data source: 2024 official provincial education expenditure statistics from China Education Economic Information Network.

Provincial expenditure gaps establish macro regional resource context but do not directly reflect institutional AI resource access at sampled universities. Pilot survey descriptive statistics further confirm student perceptions of insufficient digital learning resources: average 7-point scale scores for campus equipment adequacy (*M* = 3.45), network stability (*M* = 3.21), and library digital resource accessibility (*M* = 3.02) all fall below the scale midpoint. Interview data supplement granular evidence of campus resource shortages.

A total of 900 questionnaires were distributed, yielding 852 raw responses. Strict data cleaning rules eliminated low-quality submissions to reduce PLS-SEM path estimation bias. A response was removed only when it met both criteria: completion time under 30 s (insufficient duration to read informed consent, screening items, and full scale) and straight-lined responses across the core Likert-scale items(uniform response patterns lacking attitudinal variance).

One hundred forty-nine invalid questionnaires were removed, leaving 703 valid samples with an overall effective response rate of 82.51%. Table 2 summarizes pre- and post-cleaning sample counts.

**Table 2:** Sample counts before and after data cleaning.

Processing stage	Sample size	Percentage of raw responses (%)	Processing description
Raw collected questionnaires	852	100.00	All returned main survey submissions
Removed invalid questionnaires	149	17.49	Submissions with completion time <30s and consistent straight-lined Likert responses
Final valid analytical sample	703	82.51	Sample used for PLS-SEM, PLSpredict, CVPAT, and MGA

#### 3.2.1 Robustness check for data cleaning

High straight-lined response removal rates necessitated robustness comparisons between the full 852-response raw dataset and the cleaned 703-sample analytical dataset. Table 3 reports key model metrics across both samples. Post-cleaning model explanatory power marginally declined, yet core path directions and relative variable effect magnitudes remained stable. HTMT, VIF, and common method bias indicators all improved after removing low-quality submissions. Data cleaning primarily mitigates noise from careless responding without altering substantive research conclusions.

**Table 3:** Model metric robustness comparison: raw sample vs. cleaned analytical sample.

Metric	Raw 852 uncleaned sample	Cleaned 703 analytical sample	Comprehensive assessment
Endogenous variable BI *R*^2^	0.788	0.720	Minor explanatory power reduction; remains high
Maximum inter-construct HTMT	0.918	0.886	Improved discriminant validity; reduced construct overlap
Maximum structural model VIF	4.240	3.095	Substantially mitigated multicollinearity risk
Harman single-factor total explained variance (%)	64.177	54.077	Reduced common method bias threat
Rank of core predictor SE effect size	Highest across full model	Highest across full model	Core substantive findings unchanged

Eliminating careless straight-lined responses only reduces systematic measurement noise rather than artificially manipulating model results. Core theoretical conclusions remain robust to sample cleaning.

### 3.3 Measures

All core constructs adopted 7-point Likert scales (1 = strongly disagree, 7 = strongly agree). Scales were adapted from established theoretical literature and revised to fit generative AI educational contexts. Classic UTAUT constructs (performance expectancy, effort expectancy, social influence, continuance intention) followed measurement frameworks from Venkatesh et al. (2003). Self-efficacy drew on Bandura’s (1977) self-efficacy theory and revised scales from Wang and Chuang (2024). Personal innovativeness items adapted AI learning measurement frameworks from Hsiao and Chang (2024) but were discarded from formal structural analysis due to poor discriminant validity in pilot testing. Perceived policy signals integrated institutional policy perception scales from Wu et al. (2012), institutional GenAI policy research by Jin et al. (2025), and Polyportis and Pachos-Fokialis (2025). Algorithmic trust adapted McKnight et al.’s (2011) technology-specific trust framework, split into three dimensions: output accuracy and reliability, system stability, and personal data privacy protection.

Table 4 summarizes all constructs, abbreviations, item counts, sample items, and foundational literature sources. Personal innovativeness items were only deployed during pilot scale purification and excluded from formal structural modeling.

**Table 4:** Construct definitions, sample items, and literature origins.

Construct	Abbreviation	Number of items	Sample item	Literature source
Performance expectancy	PE	3	Using AI educational tools improves my learning efficiency.	Venkatesh et al. (2003)
Effort expectancy	EE	3	Learning to operate AI educational tools requires minimal effort for me.	Venkatesh et al. (2003)
Social influence	SI	3	Important people (e.g., instructors) believe I should use AI educational tools.	Venkatesh et al. (2003)
Self-efficacy	SE	3	I feel confident operating AI educational tools without on-site instructor guidance.	Bandura (1977), Wang and Chuang (2024)
Continuance intention	BI	3	I plan to regularly use AI educational tools in future academic work.	Venkatesh et al. (2003)
Personal innovativeness	PI	3	I actively seek and test new academic technology tools.	Hsiao and Chang (2024), Pilot survey only, excluded from formal model
Perceived policy signals	PPS	3	My university’s policies clearly encourage student AI integration in coursework.	Wu et al. (2012), Jin et al. (2025), Polyportis and Pachos-Fokialis (2025)
Algorithmic trust	AT	3	I trust AI educational tools to generate accurate, reliable academic outputs.	McKnight et al. (2011)
Disciplinary major	Control variable	1	What category does your academic major fall into?	Demographic covariate
Open-ended questions	Supplementary data	2	What constitutes your greatest challenge when using AI educational tools?	Contextual qualitative supplement for quantitative results

### 3.4 Data analysis procedure

#### 3.4.1 Quantitative analysis

SmartPLS 4.0 executed partial least squares structural equation modeling (PLS-SEM) on cleaned main questionnaire data. Three rationales guided PLS-SEM selection:

This study prioritizes explanation and prediction of continuance intention among northwestern university students using AI educational tools, aligning with PLS-SEM’s predictive orientation.

The integrated model combines canonical UTAUT constructs and novel AI education context-specific latent variables, a framework suited to PLS structural path testing.

Questionnaire data exhibit mild non-normal distribution characteristics; PLS-SEM imposes minimal restrictive assumptions on variable distributions.

Quantitative analysis followed standardized sequential steps:

Raw data quality screening: Remove submissions with completion time <30s and uniform straight-line Likert responses, retaining 703 valid samples.

Measurement model assessment: Evaluate outer loadings, Cronbach’s *α*, composite reliability, average variance extracted (AVE), Fornell–Larcker criterion, and HTMT discriminant validity.

Structural model evaluation: Estimate standardized path coefficients, statistical significance, R^2^ explanatory power, f^2^ effect sizes, and variance inflation factors (VIF) for multicollinearity detection. Bootstrap resampling with 5,000 iterations tested path significance.

Predictive validity assessment: PLSpredict and CVPAT cross-validation were used to evaluate out-of-sample predictive performance beyond in-sample explanatory fit, following recommendations for predictive assessment in PLS-SEM (Shmueli et al., 2016; Liengaard et al., 2021).

Multi-group analysis (MGA): Compare structural path disparities across subgroups defined by AI usage experience duration.

#### 3.4.2 Multi-group analysis

MGA segmented valid respondents by self-reported AI tool usage experience. Participants who selected “never used” did not complete the full scale and were excluded from subgroup comparison. Two mutually exclusive groups were formed:

Low-experience group: Usage duration <1 month or 1–3 months (*N* = 334).

High-experience group: Usage duration 3–6 months or 6 months and longer (*N* = 369).

PLSMGA tested whether significant inter-group differences existed across all six antecedent → continuance intention paths. Heterogeneity analysis clarifies boundary conditions for the extended UTAUT model and validates generalizability of the capability-centric interpretive framework across varying AI proficiency levels.

#### 3.4.3 Common method Bias assessment

All core latent variables were captured via single-wave self-report questionnaires, creating inherent common method bias (CMB) risks. This study integrated procedural and statistical mitigation strategies.

*Procedural controls*: The survey opened with informed consent documentation explicitly stating full participant anonymity, exclusive aggregate statistical data usage, absence of personal identifiers (name, student ID, phone number), and unconditional rights to terminate participation at any stage. These design features reduce social desirability bias and evaluation anxiety.

*Statistical diagnostic procedures*:

*Harman single-factor test*: Detect whether a single latent factor accounts for excessive shared response variance.

*Full collinearity VIF inspection*: Identify multicollinearity patterns signaling latent common method variance.

*Post-hoc* random marker variable testing: Quantify marker variable explanatory power and path magnitude to assess whether CMB distorts core structural paths.

If marker variable introduction produces minimal shifts in item outer loadings while retaining consistent path direction and significance, CMB cannot fully account for observed structural relationships.

#### 3.4.4 Qualitative analysis

Responses to the open-ended survey questions were reviewed descriptively to identify recurring difficulties in students’ AI tool use and to inform the follow-up interview protocol. These open-ended responses were not coded as an independent qualitative dataset. The formal qualitative analysis reported in Section 4.5 is based on the 25 semi-structured interview transcripts.

Semi-structured interviews supplemented quantitative path interpretation, following theoretical saturation principles. Twenty-five volunteers drawn from completed main survey respondents participated in individual 30–45 min interviews. Discussion topics covered concrete AI learning workflows, core usage barriers, AI output verification and revision routines, institutional policy and instructor advocacy impacts, and future sustained AI usage attitudes. Interviewees were anonymized with unique identifiers S01–S25 for transcript citation.

Interview recordings were fully transcribed and de-identified before thematic coding in NVivo. Three coding phases were applied: open coding, thematic aggregation, and theoretical integration. Two independent researchers coded overlapping transcript segments to calculate inter-coder reliability via Cohen’s Kappa (*κ* = 0.847), indicating substantial coding consistency. Theoretical saturation was achieved after the 22nd interview; no novel thematic categories emerged from subsequent participants. Qualitative thematic results contextualize quantitative effects for effort expectancy, self-efficacy, perceived policy signals, and algorithmic trust.

### 3.5 Ethical statement

Ethical approval was obtained from the Institutional Review Board of Xi’an International Studies University (Approval No.: XISU-2026-018), where the corresponding author holds her faculty appointment and through which all human-participant data collection procedures, including questionnaire administration and follow-up semi-structured interviews, were conducted. Informed consent was obtained from all participants before data collection. Interview participants also consented to audio recording, transcription, and the anonymized use of interview data for academic research purposes.

## 4 Results

### 4.1 Common method Bias assessment

Single-wave self-report survey data carries inherent CMB risks. Three complementary diagnostic methods evaluated bias severity: Harman single-factor test, full collinearity VIF screening, and post-hoc random marker variable analysis. Table 5 summarizes all diagnostic metrics.

**Table 5:** Multiple common method bias diagnostic outcomes.

Diagnostic test type	Observed indicator	Result value
Harman single-factor test	Total variance explained by first extracted factor (%)	54.077
Harman single-factor test	Number of factors with eigenvalue > 1	3
Full collinearity VIF screening	Global maximum VIF value across all latent predictors	3.052
*Post-hoc* random marker variable test	Marker variable R^2^	0.016
*Post-hoc* random marker variable test	Marker variable adjusted R^2^	0.006
*Post-hoc* random marker variable test	Maximum absolute marker structural path coefficient	0.114
*Post-hoc* random marker variable test	Maximum marker f^2^ effect size	0.010
*Post-hoc* random marker variable test	Maximum HTMT between marker and core constructs	0.071

The Harman single-factor test yielded a primary factor explaining 54.077% of total variance, exceeding conventional 50% cutoffs and signaling non-negligible CMB risk. However, three distinct factors returned eigenvalues above one, confirming response variance is not dominated by a single common latent factor. Full collinearity maximum VIF (3.052) remained below the standard critical threshold of 5. *Post-hoc* random marker variable analysis demonstrated negligible explanatory power, minimal path magnitudes, and near-zero shared variance with core theoretical constructs. Combined diagnostic evidence confirms CMB cannot fully explain the primary structural path relationships observed in the model. Subsequent result interpretation proceeds with cautious acknowledgment of cross-sectional self-report measurement limitations, expanded further in the Limitations section.

### 4.2 Measurement model evaluation

#### 4.2.1 Reliability and convergent validity

The measurement model’s internal consistency and convergent validity were evaluated via outer loadings, Cronbach’s *α*, rho_A, composite reliability (CR), and average variance extracted (AVE). All item outer loadings fell between 0.765 and 0.943, exceeding the 0.70 minimum threshold for acceptable indicator loadings. Cronbach’s α ranged from 0.722 to 0.913; rho_A ranged from 0.725 to 0.914; composite reliability values spanned 0.844 to 0.945, all meeting the 0.70 reliability standard. Every construct’s AVE exceeded 0.50, satisfying convergent validity criteria. Social influence recorded the lowest reliability and AVE metrics but still passed minimum statistical thresholds and was retained for structural modeling. Table 6 reports full measurement model reliability and convergent validity statistics.

**Table 6:** Measurement model reliability and convergent validity metrics.

Construct	Outer loading range	Cronbach’s *α*	rho_A	Composite reliability (CR)	Average variance extracted (AVE)
PE	0.907–0.939	0.913	0.914	0.945	0.851
EE	0.867–0.906	0.859	0.86	0.914	0.78
SI	0.765–0.860	0.722	0.725	0.844	0.644
SE	0.877–0.915	0.882	0.883	0.927	0.809
AT	0.880–0.943	0.904	0.914	0.94	0.839
PPS	0.871–0.902	0.88	0.88	0.926	0.807
BI	0.891–0.930	0.893	0.894	0.933	0.824

All values meet the acceptable threshold: Cronbach’s *α* > 0.70, CR > 0.70, AVE > 0.50, outer loadings > 0.70.

#### 4.2.2 Discriminant validity

HTMT criterion assessment validated discriminant validity across all latent construct pairs. All pairwise HTMT coefficients fell below the strict 0.90 cutoff, confirming statistically distinguishable latent variables. The highest HTMT value (SE vs. BI = 0.886) neared but did not cross the threshold, indicating strong correlation between self-efficacy and continuance intention while retaining formal discriminability. Additional high-correlation construct pairs appear in Table 7. Interpretations of structural paths avoid overinterpreting bivariate correlation as definitive causal relationships.

**Table 7:** HTMT coefficients for highly correlated construct pairs.

Construct pair	HTMT value
SE – BI	0.886
SI – BI	0.871
SI – SE	0.858
SE – EE	0.847
SI – PPS	0.825
PE – EE	0.781
PE – BI	0.753
PPS – BI	0.736

All HTMT values < 0.90, indicating satisfactory discriminant validity.

### 4.3 Structural model and hypothesis testing

Preceding structural path estimation, inner model multicollinearity was screened via VIF values. All predictor VIF scores ranged from 2.047 to 3.095, well below the standard 5.0 critical threshold for severe multicollinearity. The SE → BI path carried the highest VIF (3.095), remaining within acceptable limits. Supplementary Fornell–Larcker criterion and cross-loading inspection confirmed each indicator loaded more strongly onto its target latent construct than alternative constructs. Despite high bivariate correlation between self-efficacy and continuance intention, formal discriminant validity standards were satisfied.

Bootstrap resampling with 5,000 iterations tested path statistical significance. Table 8 summarizes standardized path coefficients, standard deviations, t-statistics, *p*-values, f^2^ effect sizes, and hypothesis testing outcomes.

**Table 8:** Standardized structural path coefficients, significance, and hypothesis conclusions.

Hypothesis	Structural path	*β*	SD	*t*	*p*-value	f^2^	Outcome	Interpretation
H1	PE → BI	0.251	0.036	7.032	<0.001	0.101	Supported	Small-medium effect; instrumental value driver
H2	EE → BI	−0.086	0.039	2.225	0.026	0.010	Rejected	Trivial effect; direction contradicts theoretical expectation
H3	SI → BI	0.143	0.037	3.877	<0.001	0.029	Supported	Small effect; peer/instructor external social cues
H4	SE → BI	0.437	0.042	10.527	<0.001	0.221	Supported	Medium effect; dominant predictive construct
H5	AT → BI	0.081	0.034	2.390	0.017	0.011	Weakly supported	Trivial effect; only statistically meaningful, limited practical weight
H6	PPS → BI	0.166	0.037	4.435	<0.001	0.043	Supported	Small effect; institutional policy normative cues

SD, standard deviation; f^2^ effect size: 0.02 = small, 0.15 = medium, 0.35 = large.

Performance expectancy exerted a significant positive effect on continuance intention (PE → BI: *β* = 0.251, t = 7.032, *p* < 0.001, f^2^ = 0.101). Hypothesis H1 was supported with small-to-medium practical association strength.

Effort expectancy displayed a significant negative association with continuance intention (EE → BI: *β* = −0.086, *t* = 2.225, *p* = 0.026, f^2^ = 0.010). The directional finding contradicted H2’s positive prediction, leading to H2 rejection. The negligible f^2^ effect size rules out substantive negative practical impact; this result is interpreted as statistical evidence of dissonance between surface operational ease and hidden cognitive verification costs in generative AI learning contexts.

Social influence significantly positively predicted continuance intention (SI → BI: *β* = 0.143, *t* = 3.877, *p* < 0.001, f^2^ = 0.029). H3 received support with small practical effect size, functioning as external social reference cue.

Self-efficacy delivered the strongest significant positive predictive effect on continuance intention (SE → BI: *β* = 0.437, *t* = 10.527, *p* < 0.001, f^2^ = 0.221). H4 was strongly supported with medium effect magnitude, representing the dominant explanatory variable in the structural model.

Algorithmic trust yielded a weak statistically significant positive association with continuance intention (AT → BI: *β* = 0.081, *t* = 2.390, *p* = 0.017, f^2^ = 0.011). H5 received marginal statistical support with negligible practical effect size, limiting real-world interpretive weight.

Perceived policy signals significantly positively predicted continuance intention (PPS → BI: *β* = 0.166, *t* = 4.435, *p* < 0.001, f^2^ = 0.043). H6 was supported with small effect size, capturing institutional external normative cues.

Model explanatory power for the endogenous latent variable continuance intention (BI) reached R^2^ = 0.721, adjusted R^2^ = 0.719. The six antecedent constructs collectively explain 72.0% of variance in student continuance intention toward AI educational tools, indicating robust in-sample explanatory capacity. Cross-sectional measurement constraints prevent interpreting this R^2^ as precise long-term behavioral predictive power. Table 9 reports endogenous variable explanatory power metrics.

**Table 9:** Endogenous latent variable explanatory power.

Endogenous latent construct	*R* ^2^	Adjusted *R*^2^
BI (continuance intention)	0.721	0.719

Synthesizing path coefficients and f^2^ effect sizes, self-efficacy emerges as the model’s dominant predictor. Performance expectancy constitutes a secondary instrumental value-based driver. Perceived policy signals and social influence operate as minor external normative cues. Algorithmic trust and effort expectancy achieve statistical significance yet carry trivial practical association strengths with limited real-world interpretive relevance.

### 4.4 Multi-group analysis results

MGA tested structural path stability across student subgroups differentiated by AI usage experience duration. Respondents reporting zero prior AI experience were excluded from subgroup comparison, as they did not complete full scale measurement. The low-experience subgroup contained 334 participants; the high-experience subgroup contained 369 participants. Both subgroups exhibited strong model explanatory power: low-experience group BI *R*^2^ = 0.737, high-experience group BI *R*^2^ = 0.673. The inter-group *R*^2^ difference failed to reach statistical significance (*p* = 0.110).

Subgroup path patterns revealed consistent directional trends without statistically significant inter-group disparities at the *α* = 0.05 threshold. Self-efficacy positively predicted continuance intention in both subgroups, with stronger effect magnitude among high-experience students (low-experience *β* = 0.362; high-experience *β* = 0.514). The MGA two-tailed *p*-value for inter-group difference equaled 0.062, reflecting a marginal trend approaching significance. This pattern confirms self-efficacy’s consistent predictive validity across proficiency tiers, with amplifying effects as cumulative AI usage experience accumulates.

Effort expectancy displayed non-significant negative association within the low-experience subgroup (*β* = −0.056) and statistically significant negative association within the high-experience subgroup (*β* = −0.115). Though inter-group difference remained non-significant (MGA *p* = 0.445), this within-group contrast suggests advanced users more readily perceive hidden cognitive burdens of prompt refinement, output verification, and content revision, decoupling surface operational ease from sustained usage willingness.

Algorithmic trust exerted significant positive predictive power for low-experience students (*β* = 0.133) but lost statistical significance among high-experience respondents (*β* = 0.046). Inter-group differences were non-significant (*p* = 0.214). This pattern implies a developmental shift from trust-driven initial exploration to capability-driven sustained engagement as students accumulate AI learning experience. Table 10 summarizes full subgroup path comparisons.

**Table 10:** Structural path coefficient comparison: low vs. high AI usage experience subgroups.

Structural path	Low-experience group β	High-experience group β	Directional difference	MGA two-tailed *p*-value	Statistical conclusion
AT → BI	0.133**	0.046 n.s.	Stronger in low-experience group	0.214	Non-significant inter-group gap
EE → BI	−0.056 n.s.	−0.115*	More negative in high-experience group	0.445	Non-significant inter-group gap
PE → BI	0.273***	0.175***	Stronger in low-experience group	0.148	Non-significant inter-group gap
PPS → BI	0.167**	0.187***	Near equivalent magnitude	0.800	Non-significant inter-group gap
SE → BI	0.362***	0.514***	Stronger in high-experience group	0.062	Marginal trending difference, not α = 0.05 significant
SI → BI	0.117*	0.161***	Slightly stronger in high-experience group	0.550	Non-significant inter-group gap

*p < 0.05, **p < 0.01, ***p < 0.001; n.s., non-significant at *α* = 0.05.

### 4.5 Qualitative findings

Twenty-five semi-structured interview transcripts underwent thematic analysis to contextualize quantitative structural model results. Qualitative data do not test causal hypotheses. Instead, they reconstruct real-world usage scenarios explaining core statistical path magnitudes and directional patterns. Five dominant thematic categories emerged from coding, summarized in Table 11 with respondent prevalence ratios, representative verbatim quotes, and corresponding quantitative model interpretations.

**Table 11:** Qualitative thematic coding, representative interview excerpts, and quantitative result alignment.

Core thematic category	Proportion of interviewees citing theme	Representative raw interview quotes	Corresponding quantitative model contextual explanation
Resource insufficiency: unstable campus networks, limited hardware, lack of institutional paid AI subscriptions	88% (22/25)	S05: Campus network bandwidth collapses during evening study hours; AI page loading stalls, forcing costly mobile data usage. S12: The university does not provide shared premium AI accounts. I only access ad-supported free web versions with incomplete feature sets and frequent crashes.	Document on-the-ground resource scarcity at sampled universities, explaining why students rely disproportionately on personal capacity rather than institutional infrastructure support.
Centrality of self-efficacy: mandatory manual cross-verification and revision of all AI outputs	100% (25/25)	S08: AI fabricates experimental datasets; I must recalculate all values independently for validation. S19: AI-generated code contains logical loopholes requiring manual parameter adjustment. S20: The software invents fake journal citations that demand line-by-line database cross-checking.	Validates self-efficacy’s dominant predictive weight in the structural model, operationalizing the construct as verification, error correction, and disciplinary task adaptation capacity rather than basic technical operation skill.
Surface operational simplicity paired with heavy hidden verification and adaptation cognitive burden	84% (21/25)	S03: One-click translation requires five to six prompt rewrites to fix discipline-specific grammatical errors. S22: Output generation occurs instantly, yet cross-checking and revision consume more time than original independent writing, creating severe time pressure during assignment deadlines.	Explains effort expectancy’s weak negative structural path coefficient, documenting the disconnect between superficial tool usability and unmeasured post-generation cognitive labor.
Institutional and peer recommendations provide limited reference cues, unable to substitute independent student capability	68% (17/25)	S11: Instructors only name AI tools without discipline-specific usage tutorials; all effective prompt design must be self-taught. S17: Peer prompt templates marginally reduce workload, yet full cross-verification remains individual responsibility.	Aligns with small f^2^ effect sizes for social influence and perceived policy signals; institutional advocacy functions as informational guidance rather than robust structural support.
Pragmatic skepticism toward algorithmic accuracy despite consistent auxiliary usage of AI tools	96% (24/25)	S07: AI routinely fabricates experimental data and literature citations; I never submit unmodified AI outputs for coursework. S13: I exclusively use AI for thesis outline drafting; all core content and data analysis undergo full independent verification and rewriting.	Illustrates a “pragmatic conditional usage” logic that diminishes algorithmic trust’s predictive weight: controlled manual verification decouples sustained AI tool use from unconditional algorithmic confidence.

Interview themes contextualize three pivotal quantitative results. First, every participant described mandatory manual output verification and revision workflows. Sustained generative AI learning constitutes a multi-stage judgment-correction-integration cycle rather than a single-click query task. This is consistent with the central role of SE in the structural model. Second, 84% of interviewees described the “simple interface, heavy post-processing burden” paradox, resolving the counterintuitive negative effort expectancy path: students do not perceive AI as difficult to launch, yet effective academic deployment imposes unmeasured cognitive overhead. Third, institutional policies, instructor guidance, and peer sharing only deliver informational cues without resolving core usage barriers absent structured training and shared premium AI access. Finally, nearly all respondents maintained cautious skepticism toward AI factual reliability while retaining AI as auxiliary learning infrastructure. Independent verification capacity outweighs algorithmic trust as a prerequisite for sustained engagement.

### 4.6 Supplementary predictive analysis: PLSpredict and CVPAT

PLSpredict and CVPAT cross-validation differentiated the model’s in-sample explanatory capacity from out-of-sample predictive performance. PLSpredict outcomes (Table 12) reported Q^2^ predict values above zero for all continuance intention indicator items (BI1 = 0.609; BI2 = 0.599; BI3 = 0.549), confirming baseline out-of-sample predictive correlation. PLS-SEM produced marginally lower RMSE than linear benchmark models, yet MAE metrics did not consistently outperform linear regression. The model exhibits basic predictive relevance without dominant out-of-sample forecasting advantages.

**Table 12:** PLSpredict out-of-sample predictive indicator comparison.

Observed indicator	Q^2^ predict	PLS RMSE	Linear model RMSE	PLS MAE	Linear model MAE	Composite assessment
BI1	0.609	0.842	0.856	0.622	0.618	Demonstrates baseline predictive correlation
BI2	0.599	0.838	0.847	0.631	0.639	Demonstrates baseline predictive correlation
BI3	0.549	0.893	0.896	0.664	0.652	Demonstrates baseline predictive correlation

CVPAT cross-validation loss statistics (Table 13) confirmed PLS-SEM significantly outperformed the simple indicator average benchmark model (PLS average loss = 0.736; indicator average loss = 1.778; loss difference = −1.041; *t* = 13.835, *p* < 0.001). No statistically significant predictive gap emerged between PLS-SEM and linear regression benchmarks (PLS average loss = 0.736; linear model loss = 0.751; loss difference = −0.015; *t* = 1.600, *p* = 0.110).

**Table 13:** CVPAT cross-validation loss comparison across benchmark models.

Comparison group	PLS average loss	Benchmark model average loss	Loss difference	*t*-statistic	*p*-value	Statistical conclusion
PLS vs. indicator average model (IA)	0.736	1.778	−1.041	13.835	<0.001	PLS-SEM delivers significantly superior predictive performance
PLS vs. linear regression model (LM)	0.736	0.751	−0.015	1.600	0.110	No statistically meaningful predictive difference between models

Combined supplementary predictive analysis leads to clear model positioning. The extended UTAUT framework achieves robust in-sample explanatory power (*R*^2^ = 0.721) yet only outperforms trivial average-value benchmarks in out-of-sample forecasting. No predictive advantage exists relative to basic linear regression. This study frames the model as a theoretical mechanism explanation framework for continuance intention formation under resource scarcity rather than a precise individual-level behavioral forecasting tool.

### 4.7 *Post-hoc* power analysis

A *post-hoc* power analysis was conducted based on the final analytical sample (*N* = 703) using a multiple regression noncentral F-test framework. For the overall structural model predicting BI, the effect size was calculated from R^2^ = 0.721 as f^2^ = R^2^/(1 − R^2^) = 2.584. With *α* = 0.05 and six predictors, the achieved statistical power was approximately 1.000, indicating sufficient power for detecting the overall model effect.

Additional *post-hoc* power analyses were conducted for individual structural paths using the f^2^ values reported in Table 8. With *α* = 0.05, *N* = 703, one tested predictor, and six total predictors, the achieved power was approximately 1.000 for SE → BI and PE → BI, 0.9998 for PPS → BI, and 0.9946 for SI → BI. For the two smallest effects, AT → BI and EE → BI, the achieved power was approximately 0.7930 and 0.7542, respectively. These results suggest that the model had sufficient power to detect the main positive associations, whereas the weak associations of AT and EE should be interpreted cautiously because their practical effect sizes were very small.

## 5 Discussion

### 5.1 Interpretation of Core empirical findings

#### 5.1.1 The primacy of self-efficacy

The study’s central finding identifies self-efficacy (SE) as the dominant predictor of student continuance intention toward AI educational tools. The SE → BI structural path delivered the largest standardized coefficient (*β* = 0.437, *p* < 0.001) with medium f^2^ effect size (0.221), surpassing all other antecedents by substantial margins. At resource-constrained northwestern local undergraduate universities, sustained AI learning engagement depends less on instrumental utility or interface simplicity and more on students’ confidence to independently refine prompts, cross-verify outputs, correct factual errors, and align AI materials with disciplinary assignment requirements. Recent evidence similarly suggests that students’ AI literacy and self-efficacy are closely associated with their ability to produce, evaluate, and improve AI-supported learning outcomes (Bećirović et al., 2025).

This result aligns with foundational self-efficacy theory (Bandura, 1977). Self-efficacy shapes persistence and task investment amid complex academic challenges. Generative AI’s inherent hallucination risks amplify this psychological mechanism: tools rapidly generate draft content yet regularly produce fabricated citations, factual misstatements, and logically inconsistent arguments (Ji et al., 2023). All interviewees mentioned at least one form of manual verification or output revision, suggesting that AI-assisted learning is not a passive one-step process but an iterative process of judgment, correction, and synthesis. The capability-centric interpretive perspective does not invalidate canonical UTAUT predictors. It highlights personal capability as a central explanatory factor when limited institutional resources and unreliable AI outputs co-occur.

Academic resilience literature further contextualizes self-efficacy’s primacy. Cassidy (2015) documented academic self-efficacy as a critical predictor of student coping capacity amid educational adversity. Northwestern local undergraduate students face compounded contextual stressors: unstable campus networks, restricted premium AI functionality, inadequate formal AI training, and inconsistent AI output reliability. Within this constrained environment, self-efficacy transcends generic technical confidence. It operationalizes resilience-driven capacity to independently resolve operational, evaluative, and revisionary AI learning barriers without comprehensive institutional support.

#### 5.1.2 The effort expectancy paradox

The result for EE differed from the classic UTAUT prediction. Standard UTAUT logic assumes that easier-to-use technologies are more likely to generate favorable usage intentions (Venkatesh et al., 2003). In this study, EE showed a weak but statistically significant negative association with BI (*β* = −0.086, *p* = 0.026), leading to the rejection of H2. Given the negligible f^2^ effect size (0.010), this result should not be interpreted as evidence of a strong negative effect. Rather, it suggests that ease of use may operate differently in generative AI learning contexts.

Generative AI tools may involve a dual-layer usability structure: surface-level ease of interaction combined with hidden cognitive demands after outputs are generated. Natural language interfaces can lower the initial threshold for use, but academic application often requires factual verification, citation checking, prompt revision, and disciplinary adaptation of generated outputs. Sergeeva et al. (2025) similarly suggested that students’ acceptance of generative AI cannot be fully explained by traditional technology acceptance variables and should be interpreted with attention to contextual risks and burdens. In the interviews, 84% of respondents described a gap between easy initial interaction and time-consuming post-generation checking and revision. Because EE in this study was measured with traditional ease-of-use items, the negative EE path should be interpreted as an indirect statistical signal rather than direct evidence that cognitive cost was quantitatively measured.

Cognitive load theory helps contextualize this result. Sweller (2010) noted that learning-related cognitive load includes not only task complexity but also additional information-processing demands. In the present context, generative AI’s conversational interface may reduce initial operational effort while introducing additional demands related to verification, prompt iteration, and error correction. Jose et al. (2025) similarly described the cognitive paradox of AI in education, in which AI may reduce some forms of friction while creating new evaluative pressure. Therefore, the weak negative EE path does not refute UTAUT logic. Instead, it highlights a measurement limitation: traditional ease-of-use scales may not fully capture the hidden cognitive work involved in responsible academic use of generative AI.

#### 5.1.3 Marginal effects of algorithmic trust and external institutional/social support

Algorithmic trust, perceived policy signals, and social influence all exerted statistically significant predictive effects yet carried small-to-trivial practical association strengths. Algorithmic trust yielded weak positive prediction (*β* = 0.081, *p* = 0.017, f^2^ = 0.011). Perceived policy signals and social influence produced minor effect sizes (PPS: *β* = 0.166, f^2^ = 0.043; SI: *β* = 0.143, f^2^ = 0.029). Student sustained AI tool usage does not primarily rely on unconditional algorithmic faith, institutional policy advocacy, or peer normative pressure.

Qualitative interview data corroborate this pattern. Ninety-six percent of interviewees maintained deliberate skepticism toward AI factual outputs while retaining AI as auxiliary learning infrastructure. Students adopt a pragmatic conditional usage framework: AI functions as drafting and brainstorming support without authoritative factual weight. Institutional policy and peer guidance deliver normative legitimacy and preliminary informational cues but fail to drive sustained engagement absent concrete training, shared premium AI access, and discipline-specific usage frameworks. Institutional GenAI policy research reinforces this conclusion: superficial advocacy statements fail to shift student behavior without structured training, clear ethical boundaries, and actionable disciplinary guidance (Jin et al., 2025; Polyportis and Pachos-Fokialis, 2025).

Algorithmic trust’s minimal predictive weight does not negate trust’s theoretical relevance to AI learning. Instead, it reflects calibrated cautious trust rather than blind algorithmic reliance. Leichtmann et al. (2023) documented that explainable AI systems foster balanced, calibrated trust instead of unregulated dependence. Study participants acknowledged AI instrumental value while retaining consistent skepticism, mitigating trust’s direct impact on continuance intention through habitual manual verification workflows. Sustained AI engagement emerges from a hybrid logic of limited conditional trust paired with independent corrective capacity, rather than unreserved algorithmic confidence.

### 5.2 Usage experience heterogeneity: MGA supplementary insights

Multi-group subgroup analysis identified descriptive usage experience disparities without statistically significant inter-group path differences at *α* = 0.05. Interpretations remain exploratory supplementary context rather than formal moderating effect conclusions.

First, self-efficacy retained consistent positive predictive power across both experience tiers, with amplified magnitude among high-experience students (low *β* = 0.362; high *β* = 0.514). The marginal *p*-value of 0.062 suggests a near-significant upward trend with cumulative usage. Novice student self-efficacy reflects generic technical confidence; advanced users’ self-efficacy embodies field-tested verification, correction, and disciplinary adaptation capacity refined through repeated AI learning workflows.

Second, effort expectancy’s negative association only reached statistical significance within the high-experience subgroup (*β* = −0.115, *p* < 0.05), remaining non-significant for novices. Advanced users accumulate direct experience with hidden post-generation cognitive burdens, decoupling surface interface simplicity from willingness to sustain consistent AI investment.

Third, algorithmic trust only significantly predicted continuance intention among low-experience respondents (*β* = 0.133, *p* < 0.05), losing statistical significance for high-experience students (*β* = 0.046, n.s.). This developmental pattern suggests a usage lifecycle shift: initial exploratory AI engagement relies on baseline algorithmic confidence, while long-term sustained practice hinges on self-sufficient verification capacity rather than trust in model accuracy.

### 5.3 Theoretical implications

This study delivers three distinct theoretical contributions to generative AI technology acceptance and UTAUT extension literature.

First, the research expands UTAUT’s contextual generalizability to resource-constrained higher education generative AI environments. Traditional UTAUT frameworks prioritize instrumental utility, interface simplicity, and social normative pressure. This analysis establishes self-efficacy as the dominant predictive construct, demonstrating generative AI academic adoption constitutes primarily a content judgment, verification, and adaptation capability challenge rather than a basic technical operation barrier.

Second, the study refines and operationalizes discipline-specific generative AI self-efficacy. The construct moves beyond generic technical operation confidence to encompass a multi-dimensional workflow capacity: prompt engineering, factual error detection, cross-source output verification, and disciplinary task alignment. These refined operational definitions provide actionable guidance for future AI self-efficacy scale development and validation.

Third, this work proposes a novel capability-centric interpretive framework for resource-constrained educational technology contexts. The framework does not displace canonical UTAUT predictors. It positions personal capability as an interpretive bridge connecting perceived tool value, institutional and social cues, and continued AI use under resource constraints.

Additional methodological theoretical contributions emerge from rigorous construct purification protocols. Pre-testing identified severe discriminant validity failure for personal innovativeness, demonstrating the necessity of HTMT screening to eliminate conceptually overlapping latent variables before structural modeling. Complementary PLSpredict and CVPAT analysis clarifies interpretive boundaries for high-R^2^ PLS-SEM models: robust in-sample explanatory power does not equate to reliable out-of-sample individual behavioral prediction, refining appropriate theoretical framing for future PLS technology acceptance research.

### 5.4 Practical institutional implications

Empirical results generate targeted actionable recommendations for local undergraduate universities across five northwestern Chinese provinces advancing generative AI educational integration amid constrained digital resources.

Institutional AI promotion priorities should shift from instrumental value marketing to structured student capability development. Rather than exclusively communicating AI’s efficiency benefits, universities should deliver lightweight discipline-specific hands-on training covering prompt design, factual cross-verification, citation auditing, code debugging, and task-specific AI adaptation workflows.

Low-cost institutional interventions mitigate resource insufficiency barriers. Universities can stabilize campus network bandwidth during peak evening study hours, compile curated open-source/free AI tool directories, negotiate limited shared premium AI subscriptions with generative AI platforms, and distribute discipline-standard prompt templates and step-by-step output verification checklists.

Institutional policy signals and instructor advocacy must transition from generic encouragement to concrete structured guidance. AI policy documentation should clearly demarcate academically permissible AI use cases, standardized AI auxiliary attribution protocols, academic integrity guardrails, and systematic output verification workflows. Instructors recommending AI tools must provide coursework-specific implementation examples rather than isolated tool names.

Universities should formalize a standardized “AI auxiliary + manual verification” academic learning norm. Classroom instruction should demonstrate canonical generative AI failure modes: fabricated citations, spurious experimental data, logical inconsistency, and disciplinary factual errors. Curricula should frame AI exclusively as brainstorming and drafting auxiliary infrastructure rather than authoritative standalone academic answer sources.

### 5.5 Limitations and future research directions

Several methodological and contextual limitations constrain generalizability of this study’s conclusions, outlining clear avenues for follow-up investigation.

First, cross-sectional self-report survey data cannot definitively establish causal directional relationships. The strong positive SE–BI correlation may reflect reciprocal causality: robust self-efficacy drives sustained AI usage, while accumulated consistent AI practice reciprocally strengthens self-efficacy beliefs. Longitudinal panel tracking or controlled experimental designs are required to disentangle temporal causal ordering.

Second, sampling exclusively targeted local undergraduate universities in five northwestern Chinese provinces. Findings cannot be directly generalized to eastern resource-rich universities, vocational colleges, or K-12 educational contexts without cross-regional comparative replication. Future multi-region multi-institutional sampling designs can test contextual boundary conditions for the capability-centric framework.

Third, resource insufficiency was operationalized as a study contextual boundary rather than a formal measured latent variable integrated into the structural model. Subsequent research can develop dedicated resource scarcity measurement scales capturing campus network stability, hardware access, premium AI subscription availability, digital library resource access, and institutional AI training provision to test formal moderating effects of resource constraints on antecedent-continuance intention paths.

Fourth, implicit generative AI cognitive cost was only interpreted post-hoc via qualitative interview data to explain effort expectancy’s counterintuitive negative path. No dedicated latent construct measured hidden verification and revision cognitive burden. Future scale development can operationalize GenAI-specific cognitive overhead to formally test the layered mechanism linking surface usability, hidden cognitive cost, and continuance intention.

Fifth, while the structural model achieves high in-sample explanatory power, PLSpredict and CVPAT results confirm limited out-of-sample predictive advantages relative to basic linear benchmarks. Subsequent mixed-methods research integrating objective platform usage log data and longitudinal behavioral tracking can strengthen individual-level sustained AI usage forecasting capacity.

Sixth, multi-source CMB diagnostic testing could not fully eliminate common method variance risk, despite evidence that shared response bias cannot fully explain core structural path relationships. All results are interpreted as correlational associations rather than definitive causal effects, consistent with cross-sectional self-report measurement limitations. Future multi-wave separated measurement designs can mitigate single-source bias threats.

## 6 Conclusion

Drawing on an extended UTAUT framework, the analysis examined students’ BI toward AI educational tools in resource-constrained undergraduate universities in Northwest China. Results showed that SE was the strongest predictor of BI, followed by PE, PPS, and SI. AT had only a weak positive association with BI, whereas EE showed a weak but significant negative association, contrary to the original hypothesis.

Overall, continued AI tool use in resource-constrained contexts appears to depend less on surface-level ease of use and more on students’ perceived ability to manage AI-assisted learning, including prompt refinement, output verification, error correction, and task adaptation. Qualitative findings further showed a gap between easy interaction with AI tools and the hidden cognitive work required after AI outputs were generated.

The findings contribute to UTAUT-based AI adoption research by advancing a capability-centric perspective, in which SE becomes especially important when institutional support and digital resources are limited. Because the evidence comes from cross-sectional self-report data and a regional sample, the results should be interpreted cautiously. Future research should use longitudinal designs, broader samples, and direct measures of resource constraints and hidden cognitive costs. For practice, universities should move beyond general AI promotion and strengthen students’ AI-related capabilities, especially verification, correction, and discipline-specific adaptation skills.

## Data Availability

The datasets generated and/or analyzed during the current study are not publicly available due to participant privacy and ethical restrictions, but are available from the corresponding author upon reasonable request. Requests to access the datasets should be directed to teresa_wan@163.com.
